# Low-dimensional attractor for neural activity from local field potentials in optogenetic mice

**DOI:** 10.3389/fncom.2015.00125

**Published:** 2015-10-02

**Authors:** Sorinel A. Oprisan, Patrick E. Lynn, Tamas Tompa, Antonieta Lavin

**Affiliations:** ^1^Department of Physics and Astronomy, College of CharlestonCharleston, SC, USA; ^2^Department of Computer Science, College of CharlestonCharleston, SC, USA; ^3^Department of Neuroscience, Medical University of South CarolinaCharleston, SC, USA; ^4^Department of Preventive Medicine, Faculty of Healthcare, University of MiskolcMiskolc, Hungary

**Keywords:** optogenetics, medial prefrontal cortex, electrophysiology, delay-embedding, nonlinear dynamics

## Abstract

We used optogenetic mice to investigate possible nonlinear responses of the medial prefrontal cortex (mPFC) local network to light stimuli delivered by a 473 nm laser through a fiber optics. Every 2 s, a brief 10 ms light pulse was applied and the local field potentials (LFPs) were recorded with a 10 kHz sampling rate. The experiment was repeated 100 times and we only retained and analyzed data from six animals that showed stable and repeatable response to optical stimulations. The presence of nonlinearity in our data was checked using the null hypothesis that the data were linearly correlated in the temporal domain, but were random otherwise. For each trail, 100 surrogate data sets were generated and both time reversal asymmetry and false nearest neighbor (FNN) were used as discriminating statistics for the null hypothesis. We found that nonlinearity is present in all LFP data. The first 0.5 s of each 2 s LFP recording were dominated by the transient response of the networks. For each trial, we used the last 1.5 s of steady activity to measure the phase resetting induced by the brief 10 ms light stimulus. After correcting the LFPs for the effect of phase resetting, additional preprocessing was carried out using dendrograms to identify “similar” groups among LFP trials. We found that the steady dynamics of mPFC in response to light stimuli could be reconstructed in a three-dimensional phase space with topologically similar “8”-shaped attractors across different animals. Our results also open the possibility of designing a low-dimensional model for optical stimulation of the mPFC local network.

## 1. Introduction

Synchronization of neural oscillators across different areas of the brain is involved in memory consolidation, decision-making, and many other cognitive processes (Oprisan and Buhusi, 2014). In humans, sustained theta oscillations were detected when subjects navigated through a virtual maze by memory alone, relative to when they were guided through the maze by arrow cues (Kahana et al., 1999). Also the duration of sustained theta activity is proportional to the length of the maze. However, theta rhythm does not seem to correlate with decision-making processes. The duration of gamma rhythm is proportional to the decision time. Gamma oscillations showed strong coherence across different areas of the brain during associative learning (Miltner et al., 1999). A similar strong coherence in gamma band was found between frontal and parietal cortex during successful recollection (Burgess and Ali, 2002). Cross-frequency coupling between brain rhythms is essential in organization and consolidation of working memory (Oprisan and Buhusi, 2013). Such a cross-frequency coupling between gamma and theta oscillations is believed to code multiple items in an ordered way in hippocampus where spatial information is represented in different gamma subcycles of a theta cycle (Kirihara et al., 2012; Lisman and Jensen, 2013). It is believed that alpha rhythm suppresses task-irrelevant information, gamma oscillations are essential for memory maintenance, whereas theta rhythms drive the organization of sequentially ordered items (Roux and Uhlhaas, 2014). Synchronization of neural activity is also critical, for example, in encoding and decoding of odor identity and intensity (Stopfer et al., 2003; Broome et al., 2006).

Gamma rhythm involves the reciprocal interaction between interneurons, mainly parvalbumin (PV+) fast spiking interneurons (FS PV+) and principal cells (Traub et al., 1997). The predominant mechanism for neuronal synchronization is the synergistic excitation of glutamatergic pyramidal cells and GABAergic interneurons (Parra et al., 1998; Fujiwara-Tsukamoto and Isomura, 2008).

Nonlinear time series analysis was successfully applied, for example, to extract quantitative features from recordings of brain electrical activity that may serve as diagnostic tools for different pathologies (Jung et al., 2003). In particular, large-scale synchronization of activity among neurons that leads to epileptic processes was extensively investigated with the tools of nonlinear dynamics both for the purpose of early detection of seizures (Jerger et al., 2001; Iasemidis, 2003; Iasemidis et al., 2003; Paivinen et al., 2005) and for the purpose of using the nonlinearity in neural network response to reset the phase of the underlying synchronous activity of large neural populations in order to disrupt the synchrony and re-establish normal activity (Tass, 2003; Greenberg et al., 2010). A series of nonlinear parameters showed significant change during ictal period as compared to the interictal period (Babloyantz and Destexhe, 1986; van der Heyden et al., 1999) and reflect spatiotemporal changes in signal complexity. It was also suggested that differences in therapeutic responsiveness may reflect underlying distinct dynamic changes during epileptic seizure (Jung et al., 2003).

The present study performed nonlinear time series analysis of LFP recordings from PV+ neurons: (1) to determine if nonlinearity is present using time reversal asymmetry and FNN statistics between the original signal and surrogate data; (2) to measure the phase shift (resetting) induced by brief light stimuli, and (3) to compute the delay (lag) time and embedding dimension of LFP data.

We investigated the response of the local neural network in the mPFC activated by light stimuli and determined the number of degrees of freedom necessary for a quantitative, global, description of the steady activity of the network, i.e., long after the light stimulus was switched off. Although each neuron is described by a relatively large number of parameters, using nonlinear dynamics (Oprisan, 2002) it is possible to capture some essential features of the system in a low-dimensional space (Oprisan and Canavier, 2006; Oprisan, 2009). One possible approach to low-dimensional modeling is by using the method of phase resetting, which reduces the complexity of a neural oscillator to a lookup table that relates the phase of the presynaptic stimulus with a reset in the firing phase of the postsynaptic neuron (Oprisan, 2013).

We recently applied delay embedding to investigating the possibility of recovering phase resetting from single-cell recordings (Oprisan and Canavier, 2002; Oprisan et al., 2003). Although techniques for eliminating nonessential degrees of freedom through time scale separation were used extensively (Oprisan and Canavier, 2006; Oprisan, 2009), the novelty of our approach is that we used the phase resetting induced by light stimulus to quickly identify similar activity patterns for the purpose of applying delay embedding technique.

## 2. Materials and methods

### 2.1. Human search and animal research

All procedures were done in accordance to the National Institute of Health guidelines as approved by the Medical University of South Carolina Institutional Animal Care and Use Committee.

### 2.2. Experimental protocol

Male PV-Cre mice (B6; 129P2 - Pval^*btm*1(*Cre*)*Arbr*∕*J*^) Jackson Laboratory (Bar Harbor, ME, USA) were infected with the viral vector [AAV2/5. EF1a. DIO. hChR2(H134R) - EYFP. WPRE. hGH, Penn Vector Core, University of Pennsylvania] delivered to the mPFC as described in detail in Dilgen et al. (2013).

Electrophysiological data were recorded using an optrode positioned with a Narishige (Japan) hydraulic microdrive. Extracellular signals were amplified by a Grass amplifier (Grass Technologies, West Warwick, RI, USA), digitized at 10 kHz by a 1401plus data acquisition system, visualized using Spike2 software (Cambridge Electronic Design, LTD., Cambridge, UK) and stored on a PC for offline analysis. Line noise was eliminated by using a HumBug 50/60 Hz Noise Eliminator (Quest Scientific Inc., Canada). The signal was band-pass filtered online between 0.1 and 10 kHz for single- or multi-unit activity, or between 0.1 and 130 Hz for local field potentials (LFP) recordings.

Light stimulation was generated by a 473 nm laser (DPSS Laser System, OEM Laser Systems Inc., East Lansing, MI, USA), controlled via a 1401plus digitizer and Spike2 software (Cambridge Electronic Design LTD., Cambridge, UK). Light pulses were delivered via the 50 μm diameter optical fiber glued to the recording electrode (Thorlabs, Inc., Newton, NJ, USA).

At the top of the recording track the efficacy of optical stimulation was assessed by monitoring single-unit or multi-unit responses to various light pulses (duration 10–250 ms). High firing rate action potentials, low half-width amplitude (presumably from PV-positive interneurons) during the light stimulation, and/or the inhibition of regular spiking units was considered confirmation of optical stimulation of ChR2 expressing PV+ interneurons. The optrode was repositioned along the dorsal ventral axis if no response was found. Upon finding a stable response, filters were changed to record field potentials (0.1–100 Hz). Two different optical stimulations were delivered: (1) a 40 Hz 10-pulse train that lasted 250 ms with 10 ms pulse duration followed by a 15 ms break, and (2) a single pulse with 10 ms duration. In both cases, the recording lasted for 2 s from the beginning of optical stimulus. Local field potential (LFP) activity was monitored for a minimum of 10 min while occasionally stimulating at 40 Hz to ensure the stability of the electrode placement and the ability to induce the oscillation. Additionally, LFP activity was monitored as a tertiary method of assessing anesthesia levels. Several animals were excluded from analysis due to fluctuating levels of LFP activity that resulted from titration of anesthesia levels during the experiment.

## 3. Data analysis

For each of the six animals, we analyzed 100 different trials, each with a duration of 2 s measured from the onset of a brief 10 ms stimulus until the next stimulus. For each 2 s long LFP recording, there are two regions of interest: the first approximately 0.5 s that follows the stimulus, which is the transient response of the neural network, and the last 1.5 s of the recording that is the steady activity of the network. The transient response is essential in the subsequent analysis of the steady response since it determines the amount of phase resetting induced by optical stimulus (see Section 3.2 for a detailed description of the procedure employed to determine the phase resetting induced by a light stimulus). The steady activity of the network was investigated to determine if there is any low-dimensional attractor that may explain the observed dynamics.

### 3.1. Tests for nonlinearity

Detection of nonlinearity is the first step before any nonlinear analysis. The test is necessary since noisy data and an insufficient number of observations may point to nonlinearity of an otherwise purely stochastic time series (see for example Osborne and Provencale, 1989). There are at least two widely-used methods for testing time series nonlinearities: surrogate data (Theiler et al., 1992; Small, 2005) and bootstrap (Efron, 1982). The most commonly used method to identify time series nonlinearity is a statistical approach based on surrogate data technique. The bootstrap method extracts explicit parametric models from the data (Efron, 1982).

In the following, we will only use the surrogate data method. Testing for nonlinearity with surrogate data requires an appropriate null hypothesis, e.g., that the data are linearly correlated in the temporal domain, but are random otherwise. Once a null hypothesis was selected, surrogate data are generated for the original series by preserving the linear correlations within the original data while destroying any nonlinear structure by randomizing the phases of the Fourier transform of the data (Theiler et al., 1992).

From surrogates, the quantity of interest, e.g., the time reversal asymmetry, is estimated for each realization. Next, a distribution of the estimates is compiled and appropriate statistical tests are carried out with the purpose of determining if the observed data are likely to have been generated by the process set though the null hypothesis. If the selected measure(s) of suspected nonlinearity does not significantly change between the original and the surrogate data, then the null hypothesis is true, otherwise the null hypothesis is rejected.

The number of surrogates to be generated depends on the rate of false rejections of the null hypothesis (Jung et al., 2003). For example, if a significance level of *l* = 0.05 is desired, then at least *n* = 1∕*l* = 20 surrogates need to be generated (Jung et al., 2003; Yuan et al., 2004). A set of values λ_*i*_ (with *i* = 1, …, *n*) of the discriminating statistics is then computed from the surrogates and compared agains the value λ_0_ for the original time series. Rejecting the null hypothesis can be done using: (1) rank ordering or significance testing, (2) the average method (Yuan et al., 2004), or (3) the coefficient of variation method (Theiler et al., 1992; Kugiumtzis, 2002; Jung et al., 2003).

In rank ordering, λ_0_ must occurs either on the first or on the last place in the ordered list of all values of the discriminating statistics to reject the null hypothesis (see the null hypothesis rejection using FNN Section 4.2).

In the average statistical method, a score γ (sometimes called a Z-score) is derived as follows:

$$\gamma =|\frac{\bar{\lambda }}{\lambda_{0}}-1|,$$

where $\bar{\lambda }=\frac{1}{n}\overset{n}{\underset{i=1}{\sum }}\lambda_{i}$ is the mean value of the discriminating statistics over all surrogates. If the score γ is much less than 1, then the relative discrepancy can be considered negligible. If γ is greater than 1, then the original data and the surrogates are significantly different and the null hypothesis is rejected.

In the coefficient of variation statistical method, a score γ is derived as follows:

(1) $$\begin{array}{l} \gamma =|\frac{\bar{\lambda }-\lambda_{0}}{\sigma_{\lambda }}|, \end{array}$$

where σ_λ_ is the standard deviation of the discriminating statistics over all surrogates. If the values λ_*i*_ are fairly normally distributed, rejection of the null hypothesis requires a γ- value of about 1.96 at a 95% confidence level (Stam et al., 1998; Jung et al., 2003).

For every trial and every animal we generated *n* = 100 surrogates and used two different discriminating statistics to detect potential nonlinearity in our data. The first γ score was based on the reversibility of the time series. The second discriminating statistics was based on the percentage of false nearest neighbors (see Section 4.2).

A time series is said to be reversible only if its probabilistic properties are invariant with respect to time reversal (Diks et al., 1995). Time irreversibility is a strong signature of nonlinearity (Schreiber and Schmitz, 2000) and rejection of the null hypothesis implies that the time series cannot be described by a linear Gaussian random process (Diks et al., 1995). We used the Tisean function *timerev* to compute the time reversal asymmetry statistics both for the original and the surrogate data (Hegger et al., 1999; Schreiber and Schmitz, 2000). The 100 surrogate data files for each of the 100 trials were generated using Tisean function *surrogate* (Hegger et al., 1999; Schreiber and Schmitz, 2000).

**Figure 1:**
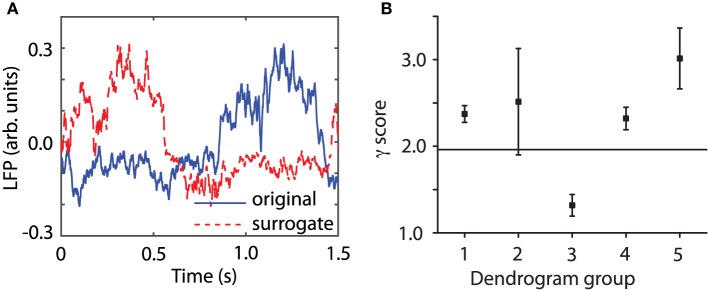
**Surrogate data**. An ensemble of 100 surrogate data sets similar to the original time series, but consistent with the null hypothesis, were generated using Tisean. **(A)** The original data from a randomly selected trial (blue continuous line) and one of its 100 surrogates (dashed red line) look similar. For each trial, 100 surrogates have been created by a stationary Gaussian linear process using the function *surrogate* of Tisean. **(B)** The discriminating statistic for the original trials and for each of their surrogates over all five groups showed that only the third group does not meet the nonlinearity criterion (the horizontal continuous line) since its γ score is less than 1.96. For all the other four groups the null hypothesis can be rejected.

Figure 1A shows one of the original time series (continuous blue line) together with one of its 100 surrogates (dashed red line). Although the two data sets might look similar, the time reversal asymmetry value for the original data was λ_0_ = 0.1893 and for the surrogate data shown in Figure 1A it was λ = 2.4948. The fact that the surrogates are significantly different from the original data means that, for example, the delay embedding dimension for surrogates is different than for the original data. Indeed, we found that the embedding dimension is higher for surrogates (see **Figure 6C**). It also means that the surrogates do not unfold correctly in the lower-dimensional embedding space of the original data (see Supplementary Materials). We used the coefficient of variation statistical method to compute a γ score from Equation (1). Figure 1B shows all γ scores for the first animal. The statistics was computed over groups of original data lumped together based on their “similarity” as determined after correcting for phase resetting induced by the light stimulus (see Section 3.2 below for details) and using the dendrogram (see Section 3.3 below for details). The average γ score of time reversal asymmetry statistics that was computed from individual λ_*i*_- values for each trial in the third group was less than 1.96. Therefore, the null hypothesis that the data had been created by a stationary Gaussian linear process could not be rejected for this group of LFPs. For all the other groups of original data formed out of the 100 trials the γ score was above 1.96 and therefore we rejected the null hypothesis. Although this time reversal asymmetry discriminating statistics seems to exclude the third group of data, we also used the FNN discriminating statistics for all data (see Section 4.2). The FNN reflects the degree of determinism in the original data and therefore serves as a good choice for a discriminating statistic (Hegger et al., 1999; Yuan et al., 2004). Briefly, for the third group of data, which was rejected based on time reversal asymmetry discriminating statistics, we found that the percentage of FNN for all 100 surrogates computed for all trials in the respective group was always larger than for the original data (see **Figure 6C**). Therefore, based on both discriminating statistics, it is likely that nonlinearity is present in all our data.

### 3.2. Phase resetting of LFP

**Figure 2:**
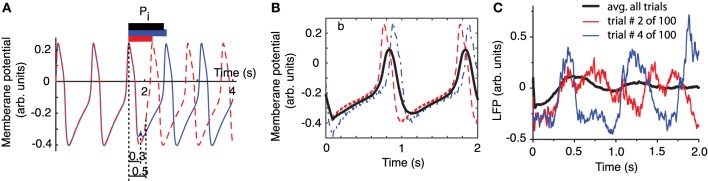
**Phase resetting of FLPs**. The free-running neural oscillator was perturbed at different phases and, as a result, its phase was reset due to a transient change in the length of the current cycle during which the perturbation was active **(A)**. The intrinsic firing period *P*_*i*_ (see black bar on top of the third cycle that contains the perturbation) was shortened by a perturbation applied at phase ϕ = 0.5 (see dashed red trace and the corresponding red bar on top of the third cycle). The same perturbation applied at phase ϕ = 0.3 (measured from the peak of the action potential—see vertical dotted lines) lengthened the current cycle (see continuous blue trace and the corresponding blue bar on top of the third cycle). **(B)** The average membrane potential of 100 noisy traces (thick black line) perturbed at 100 equally spaced phases during the third cycle is less noisy and retains some low frequency oscillations present in all individual traces. All traces were aligned at stimulus onset and only two of them are shown (red dashed and blue dashed-dotted). **(C)** LFP recordings also aligned at laser stimulus onset show an average LFP trace (black thick trace) that is almost noise free and retains some spectral characteristics of its components. At the same time, the shape of the average LFP trace is significantly different from any individual traces.

LFPs are weighted sums of activities produced by neural oscillators in the proximity of the recording electrode (Ebersole and Pedley, 2003). In order to better understand the effect of a stimulus, such as a brief laser pulse on a neural network, we used a simplified neural oscillator model (see Figure 2A) that produced rhythmic activity. We used a Morris-Lecar (ML) model neuron (Morris and Lecar, 1981). When a noise free oscillator with intrinsic firing period *P*_*i*_ (see Figure 2A) is perturbed, e.g., by applying a brief rectangular current stimulus, the effect is a transient change in its intrinsic period. For example, a perturbation delivered at phase 0.3, measured from the most recent membrane potential peak, produces a delay of the next peak of activity (continuous blue trace in Figure 2A). On the other hand, an identical perturbation delivered to the same free running oscillator at a phase of 0.5 produces a significant advance of the next peak of activity (dashed red trace in Figure 2A). As we notice from Figure 2A, the cycles after the perturbation return pretty quickly to the intrinsic activity of the cell, i.e., the most significant effect of the perturbation is concentrated during the cycle that contains the perturbation. The induced phase resetting, i.e., the permanent phase shift of post-stimulus activity compared to pre-stimulus phase, depends not only on the strength and duration of the perturbation, but also on its timing (or phase).

One approach often used for reducing the noise is averaging multiple trials. How should a meaningful average be carried out to both reduce the noise and preserve the characteristics of the rhythmic pattern, such as amplitude, phase, and frequency? One possibility is to align all action potentials at stimulus onset and added them up (see the thick black trace in Figure 2B) to generate a LFP. In Figure 2B we also added a uniform noise to neural oscillator's bias current such that the individual traces are pretty rugged. The effect of noise is especially visible on the dashes and dashed-dotted traces in Figure 2B during the slow hyperpolarization. By adding 100 noisy action potential traces produced by resetting the neural oscillator at 100 equally spaced phases we produced a smooth average (see the thick black trace in Figure 2B). Therefore, on the positive side, we could use a (weighted) sum of noisy traces to reduce the noise in our data. The other positive outcome is that the (weighted) sum retains some of the characteristics of the individual traces, such as the intrinsic firing frequency. However, we also notice form Figure 2B that the shape of the (weighted) average is quite different from any of its constituents, which raises the question: is this averaging procedure the right ways of computing a (weighted) average from individual trials? Based on Figures 2A,B, we can conclude that the mismatch between the average (black thick line) and the individual trials (blue and red traces) is due to the fact that the periodically delivered stimulus found the background oscillatory activity of the neuron at different phases, therefore, produced different phases resettings. Without correcting for the stimulus induced phase resetting effect on each trial we lose the phase and amplitude information by simply adding all individual traces. We noticed the same effects when attempting to remove the noise in out LFP data be averaging all trials aligned at the onset of the light stimulus (see Figure 2C). As a result, whenever performing an averaging of noisy rhythmic patterns for the purpose of reducing the noise, first the individual traces must be corrected for the phase resetting induced by the external stimulus.

**Figure 3:**
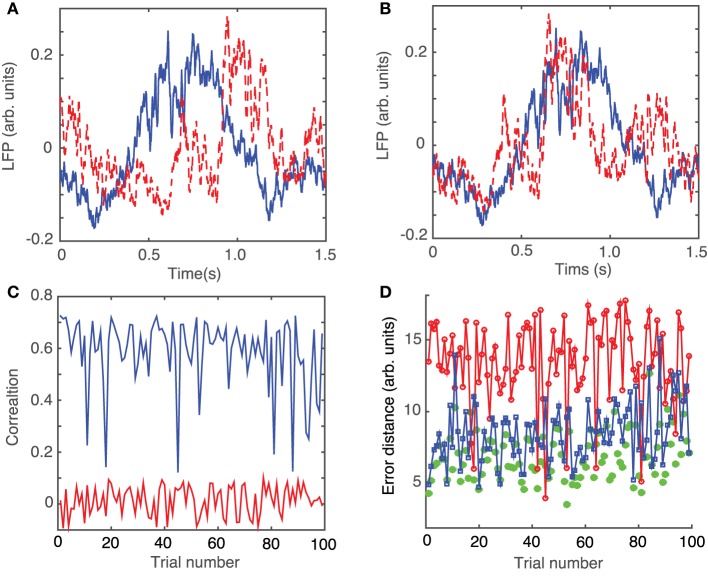
**Phase resetting correction of LFPs**. Steady LFP activity recorded 0.5 s after the 10 ms stimulus switched off look similar **(A)** and can be better overlapped by an appropriate circular shifting **(B)** that removes the waveform phase shift due to phase resetting induced by the same light stimulus arriving at different phases during the ongoing rhythm. **(C)** Without phase shifting to correct for the phase resetting, the correlations between the waveforms of different trials with respect to an arbitrarily selected “reference” trial are relatively small at an average of 0.0143 ± 0.055 (red trace). A significant improvement in pair correlation between trials occurs after appropriately shifting the waveforms to maximize the correlation coefficient (blue trace) with an average correlation of 0.5854 ± 0.1383. **(D)** Similar to correlation, the root-mean-square error between a trial and the corresponding “reference” trial significantly decreases. The rms error decreases form 13.4 ± 2.9 for correlation between trials without phase resetting correction (red line), to 8.5 ± 2.1 after phase-shifting all trials to correct for phase resetting (blue line), to 6.9 ± 1.8 for phase-shifted dendrogram-based correlation (green solid circles).

After dropping the 0.5 s transient, we noticed that even very similar LFP traces, such as those shown in Figure 3A, do not overlap perfectly due to the phase resetting (or the permanent phase shift) induced by light stimuli that arrived at different phases of the LFP activity.

In order to correct the LFP recordings for the phase resetting induced by the brief laser pulse, we performed a circular shift of each LFP trace with respect to one, arbitrarily selected, trace that was considered as a “reference” LFP. The phase resetting maximized the coefficient of correlation between any trial and the arbitrary “reference” (see Figure 3B). As a result of the circular shift, the coefficient of correlation increased significantly from an average of 0.0143 ± 0.055 (red trace in Figure 3C) to 0.5854 ± 0.1383 (blue trace in Figure 3C). Additionally, the root-mean-square (rms) error, i.e., the Euclidian norm of the difference between each 1.5 s long trial and the “reference” trial, was computed (see Figure 3D). The rms error before circularly shifting the trials was 13.4 ± 2.9. By circularly shifting the trials to remove the effect of phase resetting induced by the light stimulus, we were able to decrease the rms error to 8.5 ± 1.8 (see green curve with squares in Figure 2D).

### 3.3. Dendrograms of phase shifted LFPs

**Figure 4:**
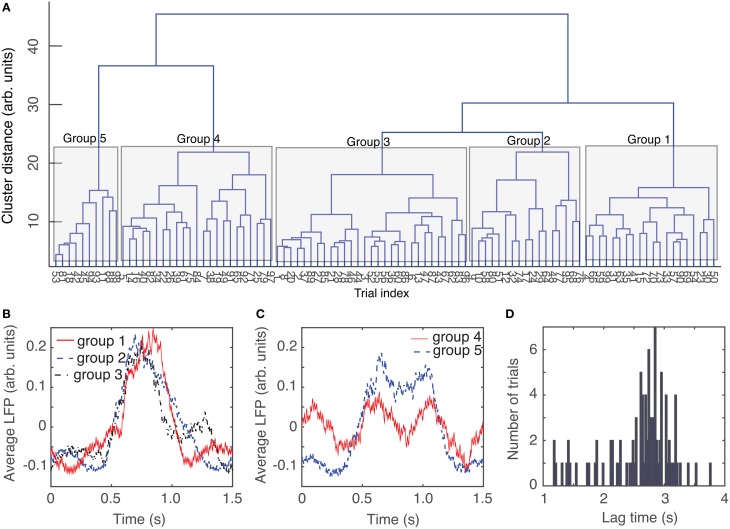
**Dendrogram-based grouping of similar LFPs**. Even after maximizing the correlation between trials by phase-shifting them to correct for phase resetting effect, the distance to an average LFP “template” can be further improved by grouping LFP waveforms according to their Euclidian-based dendrogram **(A)**. Depending on the cutoff threshold for the distance along a tree, we can have a very coarse representation with only one group (for distance larger than 40) or five groups for distance around 20 (see the shaded areas that mark different groups). As the dendrogram suggests, the average LFPs for the first three groups are quite similar **(B)**. The average LFP for the last two groups are also very similar **(C)**, but quite different from the previous three groups. The resulting rms error of a trial with respect to its corresponding group average is definitely an improvement over the simple averaging of all trials (see Figure 3D). Even though the group average could be pretty close to capturing features of individual LFP trials form the respective group, the delay time for phase space reconstruction has a wide range of values **(D)** and there is no obvious group correlation.

The circular shift performed in the previous section with the purpose of maximizing the coefficient of correlation between any trial and an arbitrary “reference” helps correctly defining the relative phase of trials with respect to each other. Another helpful step in the process of automatic data classification before attempting a delay embedding reconstruction was to separate the trials in “similar”-looking groups. Since we were interested in finding out if there is any attractor of network's steady activity, it is expected that phases space traces of different trials would remain close to each other at all times. This implies that individual recordings present some “similarities” that could be detected using the dendrograms, e.g., for the purpose of separating clean data from artifacts (due to malfunction of laser trigger, etc.) We used dendrograms to find the similarity trees of all 1.5 s long, phase-corrected, trials that allowed us to further decrease the rms error to an arbitrarily selected “reference” from the same group (see blue solid circles in Figure 3D). The dendrogram in Figure 4A used the Euclidian distance to measure similarities between the phase-shifted LFP trials.

The dendrogram could be used, for example, to separated groups of trials based on an arbitrary selection of the cutoff distance along dendrogram's trees. For example, by selecting a cluster distance larger than 40 (see Figure 4A) all 100 trials belong to just one group. As already discussed, lumping all trails in one group may inadvertently lump together low-dimensional attractors with data affected by various equipment malfunctions. Such an approach would make the task of identifying any phase space attractor to which all trajectories remain close at all times more computationally intensive. By decreasing the cluster distance threshold, we could form two groups or more. In the following, we used a cutoff cluster distance close to 20 and obtained five dendrogram-based groups (see the shaded rectangles in Figure 4A). The plots of the LFPs for each of the first three groups (Figure 4B) show pretty similar waveforms and quite different form the last two groups of the dendrogram (see Figure 4C). Therefore, it may be easier to visually identify an attractor (if one exists) by looking at reconstructed attractor of individual trials from the same group, for example by comparing traces from group 1 against each other (see **Figure 7B1**). The same is true when comparing trials from group 5 against each other (see **Figure 7B5**). It is unlikely that we would be able to find any trials from group 1 that remain close to any trials from group 5, a fact that we learned during data preprocessing stage using dendrograms.

The same numerical procedure was applied to all data from six animals of which we only show one detailed example.

## 4. Delay embedding method

Given the complexity of a single pyramidal neuron and the intricacy of synaptic coupling in the mPFT cortex (Schnitzler and Gross, 2005), we would expect a rather high-dimensional delay embedding for our LFP recordings.

In electrophysiology, we record the membrane potential time series, which is just one of many independent variables required for a full characterization of neural network activity. Even though we have direct access to only one variable of the *d*−dimensional dynamical system, i.e., the light-activated local network, it is still possible to faithfully recover, or reconstruct, the phase space dynamics through delay embedding method (Abarbanel, 1996; Kantz and Schreiber, 1997; Schuster and Just, 2005; Kralemann et al., 2008). For a time series *x*_*i*_ = *x*(*iΔt*) with *i* = 1, 2, …, *N* where *N* is the number of data points and Δ*t* is the (uniform) sampling time, a *d*−dimensional embedding vector is defined as

$$x_{i}=\left(x_{i},x_{i+n},\ldots ,x_{i+\left(d-1\right)n}\right),$$

where τ = *nΔt* is the delay, or lag, time (Packard et al., 1980; Takens, 1981).

Two parameters are essential for a correct delay embedding reconstruction of the phase space: the lag time τ and the embedding dimension *d*_*E*_. The delay, or lag, time τ is the time interval between successive components of the embedded vector. Although we assumed that the same delay time applies to each component of the embedded vector, the delay embedding method also allows for different delays along different directions of the phase space (Vlachos and Kugiumtzis, 2010).

### 4.1. Lag time

The quality of phase space reconstruction is affected, among other factors, by the amount of noise, the length of the time series, and the choice of the delay time. For example, a too small delay time τ leads to embedded vector with highly correlated, or indistinguishable, components. Geometrically, this means the all trajectories are near the diagonal of the embedding space and the attractor has a dimension close to one irrespective of its complexity. To avoid such *redundancy*, the delay time τ should be large enough to make the components of the embedded vector independent of each other. However, a too large delay time completely de-correlates the components of the embedded vector. Geometrically, this means that phase space points fill the entire embedding space randomly and the attractor has a dimension close to the embedding space dimension. Although there is no universal method for selecting the “right” delay time, in practice we use a few different approaches to avoid both the *redundancy* due to a too short delay time and the *irrelevance* due to a too large delay time (Casdagli et al., 1991).

One of the methods often used for estimating the lag time τ is the *autocorrelation* of the time series. Although researchers agree that autocorrelation could provide a good estimation of the time lag, there is no consensus regarding the specifics. For example, Zeng et al. (1991) considered that τ is the time at which the autocorrelation decays to *e*^−1^, Schiff and Chang (1992) considered the first time when the autocorrelation is not significantly different from zero, Schuster (Schuster and Just, 2005) suggested using the first zero of autocorrelation function to ensure linear independence of the coordinates, King et al. (1987) considered the time of the first inflection of the autocorrelation, and Holzfuss and Mayer-Kress (1986) considered the first time the autocorrelation reaches a minimum.

In addition to autocorrelation, Fraser and Swinney (1986) suggested using the first local minimum of *the average mutual information* (AMI) to estimate the time lag. Their method measures the mutual dependence between *x*_*i*_ and *x*_*i* + *n*_ with variable lag time *nΔt* (see also Kantz and Schreiber, 1997; Hegger et al., 1999).

Additionally, the total time spanned (Broomhead and King, 1986) by each embedded vector, i.e., *t*_*w*_ = (*d* − 1)τ, is a significant measure of potential crossover between temporal correlation that could induce spurious spatial, or geometrical, correlation between phase space points (Theiler, 1990).

### 4.2. Embedding dimension

The embedding dimension was selected based on Takes's theorem (Takens, 1981) that ensured a faithful reconstruction of a *d*−dimensional attractor in an embedding space with at most 2*d* + 1 dimensions. For a dissipative system, Hausdoff dimension could be estimated from a time series and used as the dimension of the attractor (Holzfuss and Mayer-Kress, 1986; Kennel et al., 1992; Provenzale et al., 1992). Good estimators of Hausdorf's dimension are the correlation dimension (Grassberger and Procaccia, 1983) or the Lyapunov dimension (Kaplan and Yorke, 1979). Once the range (*d* ≤ *d*_*E*_ ≤ 2*d* + 1) of embedding dimensions is known, additional tests could determine the optimum embedding dimension *d*_*E*_.

Kennel et al. (1992) introduced the false nearest neighbors (FNN) procedure to obtain the optimum embedding dimension (see also Kennel et al., 1992; Hegger et al., 1999; Sen et al., 2007). The idea behind FNN approach is to estimate the number of points in the neighborhood of every given point for a fixed embedding dimension. High dimensional attractors projected onto a too low dimensional embedding space show a significant number of false neighbors, i.e., phase space points that look close to each other although in the true attractor space they are far apart. The FFN method compares the Euclidian distance *R*_*d*_ between two neighbors *x*_*i*_ and *x*_*j*_ computed in a *d*−dimensional space against the distance *R*_*d* + 1_ in a (*d* + 1)−dimensional embedding space (Kennel et al., 1992). If the ratio of relative distances between neighbors in the two embedding spaces, i.e., $f=\sqrt{\frac{R_{d+1}^{2}-R_{d}^{2}}{R_{d}^{2}}}$, is larger than a predefined value then the two points *x*_*i*_ and *x*_*j*_ are false neighbors, i.e., the points are neighbors because of a too low projection and not because of the true dynamics. The ratio *f* is usually set between 1.5 and 15 (Kennel et al., 1992; Abarbanel, 1996; Kantz and Schreiber, 1997). Additionally, if the distance *R*_*d* + 1_ is larger than the coefficient of variation $\sigma ∕\bar{x}$ of the data then the two points are false neighbors. The reason is that σ is a measure of the size of the attractor and two points that are false neighbor will be indeed stretched to the extremities of the attractor in dimension *d* + 1. Abarbanel (1996) found that for many nonlinear systems the value of *f* approaches 15, but the range is quite wide from 9 to 17 (Konstantinou, 2002). By successively computing the fraction of FNNs in different embedding dimensions, it is possible to estimate an optimum embedding. Some algorithms that takes into account the temporal window *t*_*w*_ = (*d* − 1)τ spanned by the embedded vectors allow simultaneous estimation of both embedding dimension and lag time (see Stefánsson et al., 1997).

## 5. Results

### 5.1. Experimental data

Since we were interested in uncovering any possible attractor of phase space trajectories, we only considered the last 1.5 s of each 2 s long recording. We first performed a phase shift of every 1.5 s long LFP recording to correct for the phase resetting due to light stimulus (see Figure 3B for two similar-looking LFT traces that were phase-shifted with respect to each other to maximize the correlation coefficient and correct for the phase resetting effect).

### 5.2. Lag time

As described in Section 4.1, we used two different approaches to estimating the lag time τ: (1) the autocorrelation function (Casdagli et al., 1991), and (2) the AMI method (Fraser and Swinney, 1986). The first zero crossing of the autocorrelation function is the time τ beyond which *x*(*t* + τ) is completely de-correlated from *x*(*t*). However, the first zero crossing of the autocorrelation function takes into account only linear correlations of the data (Abarbanel, 1996). The first minimum of the nonlinear autocorrelation function called *Average Mutual Information* (AMI) (Fraser and Swinney, 1986) is considered a more suitable choice since this is the time when *x*(*t* + τ) adds maximum information to the knowledge we have from *x*(*t*) (Kantz and Schreiber, 1997). In most practical applications the two methods are used together and they usually give similar estimations of the lag time.

We computed the lag times for individual trials (see Figure 4D for the distribution of all lag times for animal # 1) and also for group averages (see Table 1). Although only the autocorrelation-based lag time are shown both in Figure 4D and Table 1, the AMI-based lag time values (not shown) were within 10% of those obtained with the autocorrelation.

**Table 1:** **Estimated lag times**.

**Mouse #**	**Avg**.	**Std**.	**Group 1**	**Group 2**	**Group 3**	**Group 4**	**Group 5**
1	2599	542	1504	2962	2886	2578	2721
2	3150	885	3128	2982	4337	3390	3401
3	1759	483	2297	1814	1812	2203	
4	2645	708	3394	2924	2611	3332	
5	1842	708	1501	1722	1717	2286	
6	1661	594	1518	1767	1583	1736	1374

In Table 1, the second column (called Avg.) and the third columns (called Std.) represent the average, respectively, the standard deviation of the corresponding lag time distributions, such as the one shown in Figure 4D for animal # 1. The next columns in Table 1 represent the lag times of the dendrogram-based group averages.

**Figure 5:**
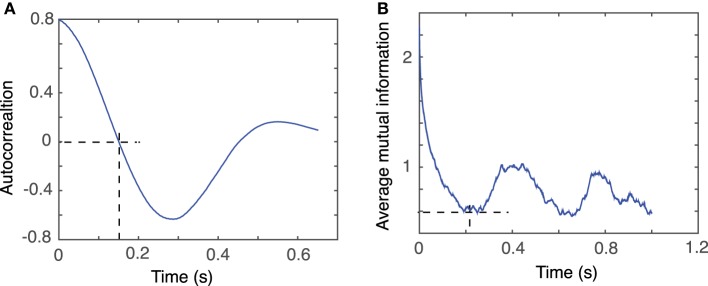
**Time lag estimation**. The first zero crossing of autocorrelation function is around τ ≈ 1500Δ*t* **(A)** and the first minimum of the average mutual information is around τ ≈ 2000Δ*t* **(B)** with Δ*t* = 10^−4^ *s*.

For example, for the first animal, the first zero crossing of the autocorrelation function for dendrogram-based average LFP of group 1 is around τ ≈ 1500Δ*t* (see Figure 5A), whereas the first minimum of the AMI is around τ ≈ 2000Δ*t* (see Figure 5B).

Our data were stored as single-column text files representing the LFP recordings with a sampling rate of Δ*t* = 10^−4^ *s*. Tisean command for estimating the lag time from autocorrelation function was *autocor dataFile.txt -p -o*, where the option −*p* specified periodic continuation of data and −*o* specified that the expected output will be returned to a file named *dataFile.txt.co*, which is plotted in Figure 5A.

Tisean command for estimating the lag time from the AMI was *mutual dataFile.txt -D10000 -o*, where the option −*D*10000 specified the range of lag times for which AMI was computed and stored in the file *dataFile.txt.mut*, which is plotted in Figure 5B.

### 5.3. Embedding dimension

**Figure 6:**
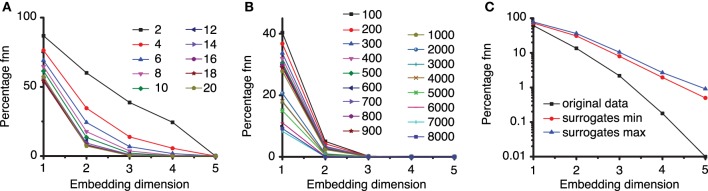
**Percentage of false nearest neighbors. (A)** For a too small ratio *f* < 7 of distances between neighbor points in different embedding dimensions, the percentage of false nearest neighbors is high and only drops near zero for very large embedding dimensions. For larger ration *f* > 7 all percentages drop to almost zero false nearest neighbors for an embedding dimension of *d*_*E*_ = 3. This suggests that an optimum ratio is above *f* = 7, in agreement with results from others (Abarbanel, 1996; Konstantinou, 2002). **(B)** To avoid spurious spatial correlations due to inherent temporal correlation between too closely spaced points in a time series, the percentage of FNN was estimated with variable Theiler window (*t*). **(C)** The percentage of FNN is also a good discriminating statistics. For the third group of data from the first animal, the logarithmic plot shows that the percentage of FNN for the original data (solid squares) is always smaller than any of the 100 surrogates. Only the envelopes of the minimum (solid circles), respectively, maximum (solid triangles) values of FNN are shown.

The method of false nearest neighbors (FNN) estimates the embedding dimension *d*_*E*_ by repeatedly increasing the embedding dimension until the orbits of the phase space flow do not intersect or overlap with each other. We used a lag time τ = 2200Δ*t* and estimated the embedding dimension using FNN method with ratios *f* between 2 and 20 (see Figure 6A). As expected, for large ratios of distances, e.g., *f* > 7, the percentage of FNNs drops to almost zero for an embedding dimension *d*_*E*_ = 3.

The actual Tisean routine used was *false_nearest dataFile. txt -f2 -d2200 -o*, which calculated the percentage of FNNs with a ratio *f* ≥ 2, a lag time *d* = 2200Δ*t*, with the default phase space dimensions from 1 to 5 (see Figure 6A). Figure 6A clearly indicates that an embedding dimension *d*_*E*_ = 3 is sufficient.

Any estimate of dimension, especially when it is based on correlation among data points, assumes that pairs of points are drawn randomly and independently according to the scale invariant measure of the attractor. However, points occurring close in time are not independent and lead to spuriously low estimates of embedding dimension. To avoid this issue, points closer than some minimum time (called the Theiler window) can be excluded from calculations (Grassberger, 1987; Theiler, 1990). Heuristic examples of estimates of Theiler window are three times the correlation time (Heath, 2000), (*d* − 1)τ, or other *ad hoc* values based on space-time separation plots (Provenzale et al., 1992).

In our estimation of embedding dimension with FNN method, we also tested a wide range of Theiler windows from 100 to 8000 sampling times (Figure 6B) in order to make sure that no spurious temporal correlation among data points led us to a too low estimation of the embedding dimension. All plots of the fraction of FNNs indicated that *d*_*E*_ = 3 is still a good choice of the embedding dimension. The actual Tisean routine was *false_nearest dataFile.txt -f20 -d2200 -t100 -o*, which calculates the percentage of FNNs with a ratio greater than *f* = 20, a lag time of *d* = 2200Δ*t*, a Thriller windows −*t* of 100Δ*t* for all embedding dimensions from 1 to 5 (see Figure 4B).

**Figure 7:**
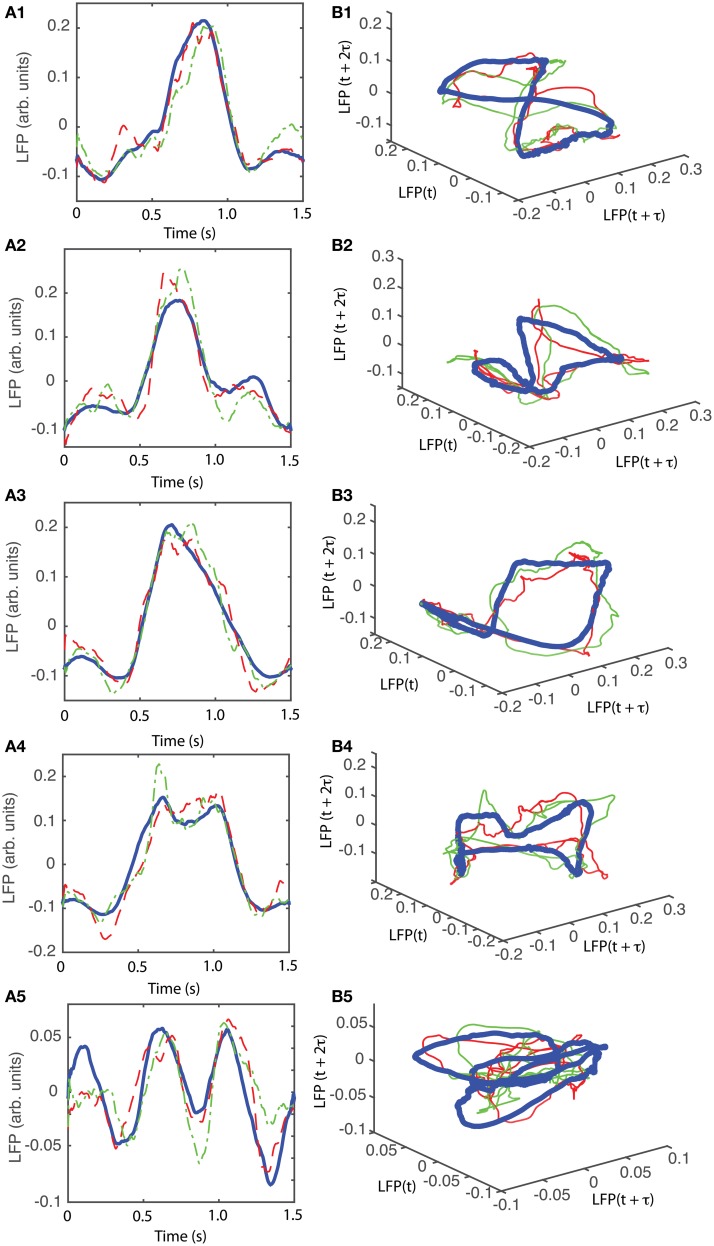
**Reconstructed 3-dimensional attractor for average activity of the local network**. From each 2 s long trial only the last 1.5 s of the steady LFP recording was used. **(A1–A5)** Show the average LFP for each of the five groups of the corresponding dendrogram (blue thick line) as a visual aid to guide us gauge if the two randomly selected trials from the same group (red dashed and green dashed-dotted line) remain close to each other al all times. **(B1–B5)** Show the corresponding three dimensional reconstructed attractors. With the exception of the last group of LFP recordings, the attractors look similar after they are appropriately rotated and/or phase shifted.

**Figure 8:**
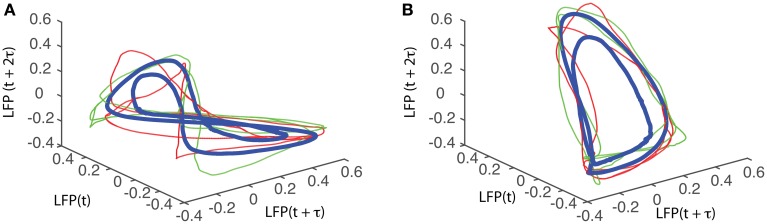
**“8”-shaped attractor and its topologically equivalents**. The same data sets were reconstructed in three dimensions with different delay times to produce topologically equivalent attractors. **(A)** A typical “8” shaped attractor made of two twisted loops can be obtained for a delay time of τ = 1900Δ*t*. Using a delay time τ = 2200Δ*t*, the attractor is “untangled” and becomes quite flat **(B)**.

The attractors were reconstructed (see Figure 7) using the time lag τ and embedding dimension *d*_*E*_ as determined above. AAs seen form Figures 7A1–A5, the dendrogram-based preprocessing separated quite well the LFP waveforms in “similar” groups such that randomly selected LFPs from the same group remained close to each other at all times (see red and green traces in Figures 7B1–B5). The reconstruction of individual trials was performed with their corresponding delay (lag) times (see Figure 4D for the distribution of all delay times for the first animal). We also showed the reconstructed group average (blue thick trace in Figures 7B1–B5) not because it represents the “true” attractor, but rather as a visual cue to help us gauge if the phase space trajectories of the individual trials remained close to each other and at all times. As expected from the dendrogram-based preprocessing, the first three groups gave very similar reconstructed attractors. The shape of attractors from the first three groups could be roughly described as a continuous circular loop twisted in an “8”-shaped object (see Figures 7B1–B3). Since the group average (blue thick line) is less noisy than the individual trials (red and green lines) it serves as a visual aid toward identifying the shape of the attractor suggested by the individual trials. The shape of the first group's attractor (Figures 7B1–B3) could be viewed as an “8”-shaped loop bent around its midpoint (see also Supplementary Materials Video). However, by increasing the lag time, the “8”-shaped attractor can be “untangled” such that the two loops look more like the circles shown in Figures 7B2,B3. For example, in Figure 8 we showed two examples of the same trials (red and green lines) together with their corresponding group average (thick black trace) that were reconstructed in the three dimensional phase space using different delay times. In Figure 8A for τ = 1900 we clearly notice the twisted “8” shaped attractor that looks straight in Figure 8B for a delay time of τ = 2200. Therefore, all attractors in Figures 7B1–B3 are topologically identical (up to some microscale details) since any of them could be morphed into another by a (circular) phase shift. Furthermore, a close inspection of the fourth's group attractor shows that it is close to the previous three and quite different from the fifth attractor.

**Figure 9:**
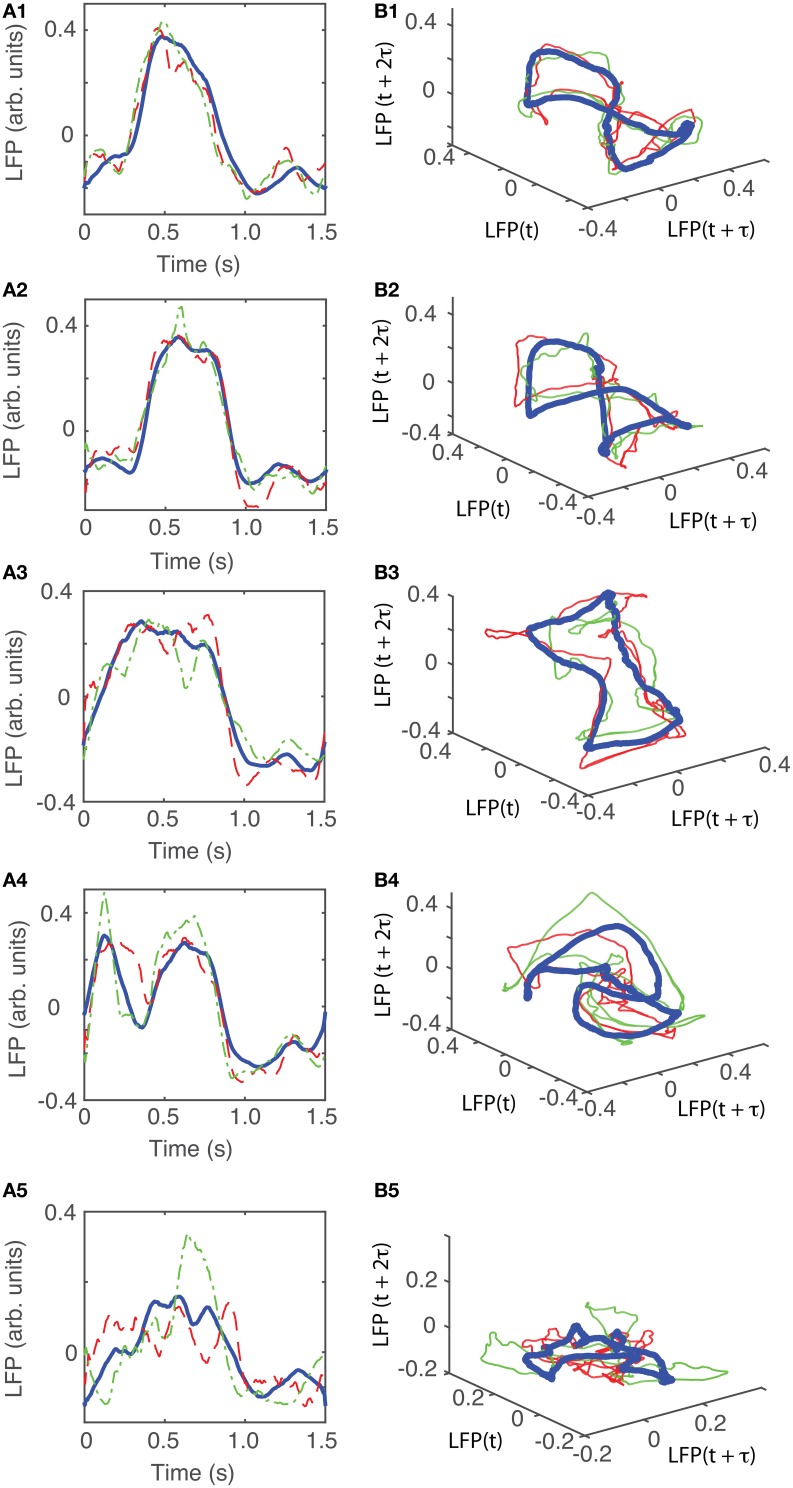
**Reconstructed 3-dimensional attractor for animal #2. (A1–A5)** Show the average LFP for each of the five main groups of the corresponding dendrogram (blue thick line) and two randomly selected trials form the same group (red dashed and green dashed-dotted line). **(B1–B5)** Show the corresponding three dimensional reconstructed attractors. With the exception of the last group of LFP recordings, the attractors look similar after they are appropriately rotated and/or phase shifted.

**Figure 10:**
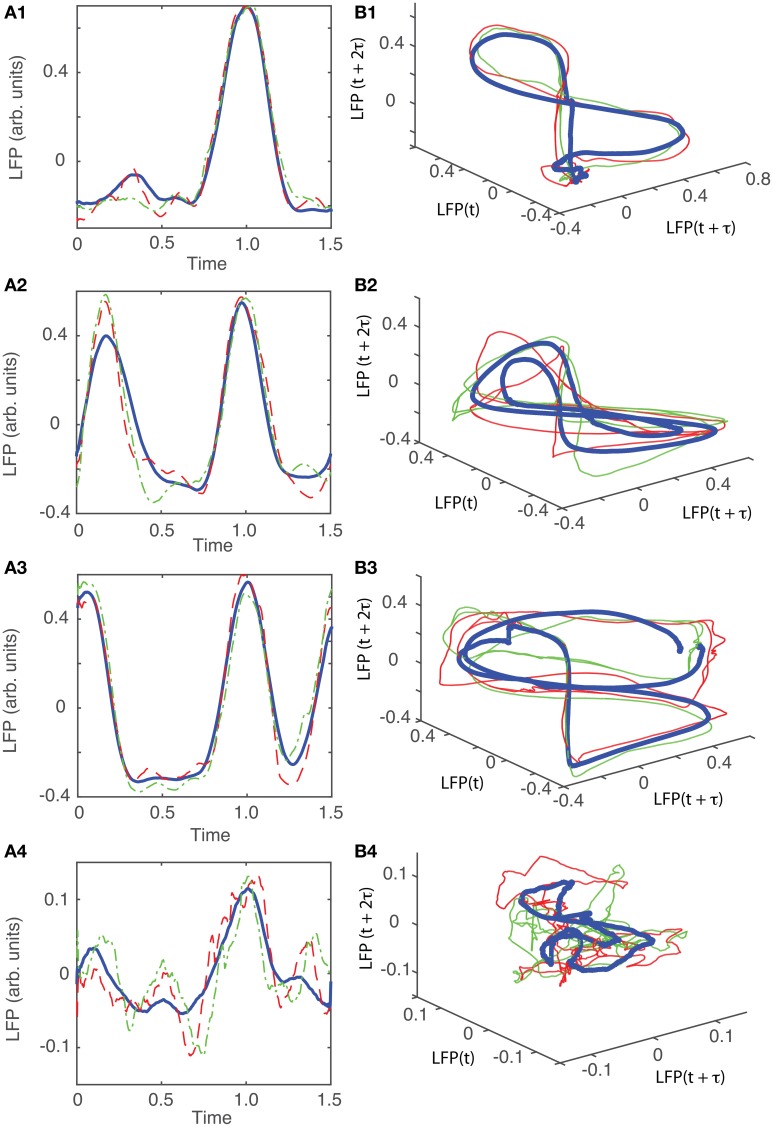
**Reconstructed 3-dimensional attractor for animal #3. (A1–A4)** Show the average LFP for each of the four main groups of the corresponding dendrogram (blue thick line) and two randomly selected trials form the same group (red dashed and green dashed-dotted line). **(B1–B4)** Show the corresponding three dimensional reconstructed attractors. With the exception of the last group of LFP recordings, the attractors look similar after they are appropriately rotated and/or phase shifted.

**Figure 11:**
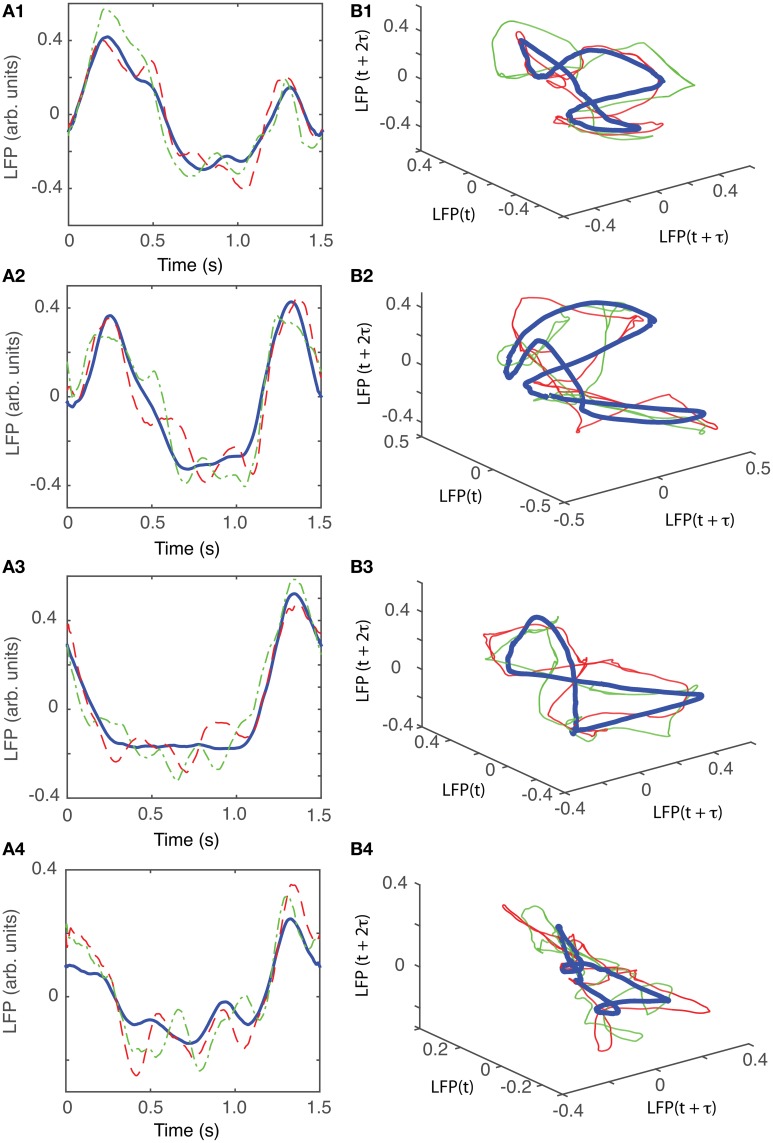
**Reconstructed 3-dimensional attractor for animal #4. (A1–A4)** Show the average LFP for each of the four main groups of the corresponding dendrogram (blue thick line) and two randomly selected trials form the same group (red dashed and green dashed-dotted line). **(B1–B4)** Show the corresponding three dimensional reconstructed attractors. With the exception of the last group of LFP recordings, the attractors look similar after they are appropriately rotated and/or phase shifted.

**Figure 12:**
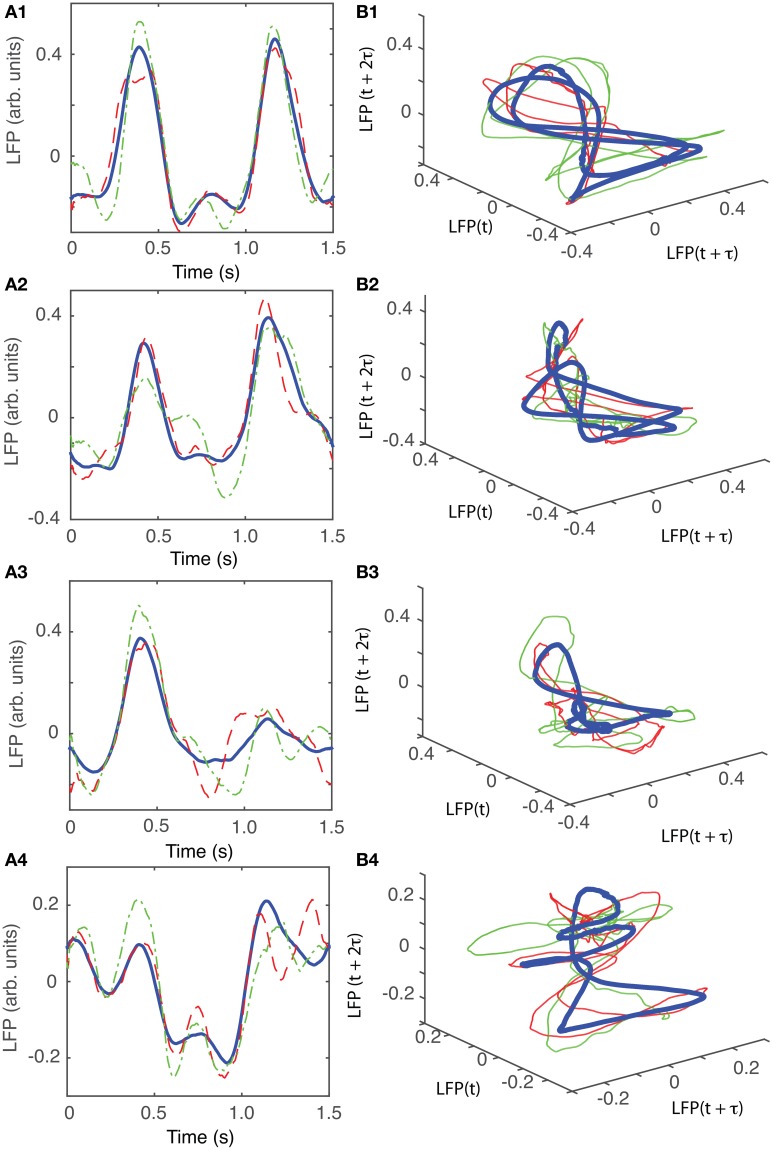
**Reconstructed 3-dimensional attractor for animal #3. (A1–A4)** Show the average LFP for each of the four main groups of the corresponding dendrogram (blue thick line) and two randomly selected trials form the same group (red dashed and green dashed-dotted line). **(B1–B4)** Show the corresponding three dimensional reconstructed attractors. With the exception of the last group of LFP recordings, the attractors look similar after they are appropriately rotated and/or phase shifted.

**Figure 13:**
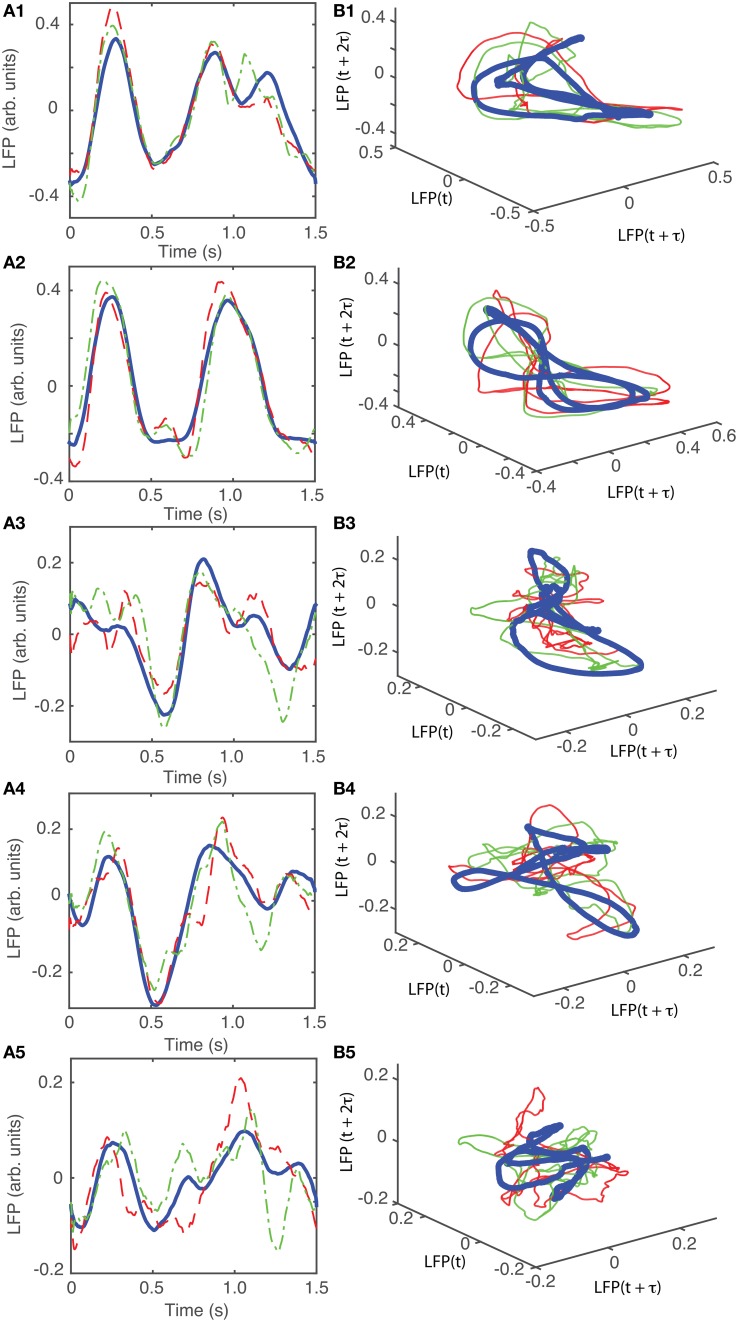
**Reconstructed 3-dimensional attractor for animal # 6. (A1–A5)** Show the average LFP for each of the five main groups of the corresponding dendrogram (blue thick line) and two randomly selected trials form the same group (red dashed and green dashed-dotted line). **(B1–B5)** Show the corresponding three dimensional reconstructed attractors. With the exception of the last group of LFP recordings, the attractors look similar after they are appropriately rotated and/or phase shifted.

The detailed procedure described above was also applied to the other five data sets from different animals. The results are summarized in Figures 9–**13**. For all six animals that were retained and analyzed, the zero crossings of the autocorrelation and the minimum the AMI gave consistent lag time estimations (see Table 1).

We found that for all six animals the optimum delay embedding dimension was *d*_*E*_ = 3. We found topologically identical attractors in all first four LFP dendrogram-based groups for animal #1 (see Figures 7B1–B4), which cover 90% of the recordings. The attractor is “8”-shaped and is topologically equivalent (after appropriate phase shifting) with an “untangled” attractor (see Figure 8).

For animal #2, all attractors belong to the same “8”-shaped class or its topologically identical counterparts (see Figures 9B1–B5), although the fifth group presented a very large variability.

For animal #3, there were three topologically identical dendrogram-based LFP groups that gave an “8”-shaped attractor (see Figures 10B1–B3), which covered 84% of recordings.

For animal #4, all attractors were topologically identical that belonged to the “8”-shaped attractor (see Figures 11B1–B4), although the fourth group presented a very large variability.

For animal #5, there were again three topologically identical dendrogram-based LFP groups that belonged to the “8”-shaped attractor (see Figures 12B1–B3), which covered 74% of recordings.

For animal #6, there were two topologically identical dendrogram-based LFP groups that belonged to the “8”-shaped attractor (see Figures 13B1,B2), which covered 34% of recordings.

An important characteristic of the attractors that were not included in the above category of “8”-shaped attractors or their topological equivalents is that all of them showed relatively low amplitude oscillations of the LFP. For example, while the peak-to-peak amplitude of LFP oscillations for the four topologically equivalent attractors shown in Figures 7B1–B4 was between −0.15 and +0.25 arb. units, the amplitude of the LFP for the last group was between −0.075 and 0.075 arb .unit., which is a decrease by a factor of 2.6. Similarly, for animal #3, the range of LFP for the “8”-shaped attractor or their topological equivalents (see Figures 10B1–B3) was between −0.4 to +0.7 arb. units whereas for the only dissimilar group the LFP amplitude was between −0.1 to +0.1, a decrease in amplitude of LFP by a factor of 5.5. For animal #5, the decrease in amplitude of LFP only by a factor of 1.5 and for animal #6 the factor was 2.5. One possible explanation could be an intermittent malfunction of the laser's trigger. The dendrogram method helped us automatically sort the data set into “similar” groups before performing a delay embedding. As a result, we decreased the computational time by eliminating pair comparisons of all reconstructed attractors to determine which trials remain close to each other.

## 6. Discussion

Accurate quantification of the dynamic structure of LFPs can provide insight into the characteristics of the underlying neurophysiological processes that generated the data. In the present study, we first determined that nonlinearity is present in our LFP data using the surrogates method and two different discriminating statistics: (1) time reversal asymmetry, and (2) percentage of FNN. Time reversal asymmetry is a robust method for detecting irreversibility, which represents nonlinearity, even in the presence of a large amount of noise in the time series (Diks et al., 1995). Time reversal asymmetry statistics revealed clear differences between the original and the surrogates, with the exception of one group of data out of five for the first animal. For each of the six animals we had one group of original data for which we could not reject the null hypothesis that the time series could be produced by a linearly filtered noise at a significance level of 5% (Stam et al., 1998).

We performed also a FNN-based nonlinearity test and found that for all LFPs the percentage of FNN is always smaller for the original data trials compared to any of their surrogates. For example, any of the individual trials from the group of data for which we could not reject the null hypothesis based on time reversal asymmetry criterion had a smaller percentage of FNN than any of its 100 surrogates (see Figure 6C). As a result, we concluded that nonlinearity is likely present in all our data sets.

We performed two important data preprocessing that helped us reduce the computational time required for attractors identification: (1) phase shifting LFPs to correct for the phase resetting induced by light stimulus, and (2) grouping the shifted LFPs in similar patterns of activity using a dendrogram (see Figure 4A).

Since the light stimulus was applied every 2 s, it found the rhythmic LFP activity at different phases. As a result, it produced significantly different permanent phase shifts of the LFPs from trial to trial (see the two out-of-phase red and blue LFP recordings in Figure 2A). We determined the amount of phase resetting by circularly shifting the recordings (for example, compare the out-of-phase traces in Figure 3A against a better overlap of LFPs in Figure 3B). The phase resetting in neural networks is of paramount importance for large neural network synchronization. For example, in deep brain stimulation (DBS) procedures an electrical pulse is applied through an electrode to a brain region with the purpose of disrupting the synchronous activity, e.g., during epileptic seizures (Varela et al., 2001; Tass, 2003; Greenberg et al., 2010). For this purpose, stimuli are carefully designed with appropriate amplitude and duration and are precisely delivered during DBS procedures (Tass, 2003; Greenberg et al., 2010). Such procedures are based on precise measurements of phase resetting. Although we did not use electrical stimuli like in DBS, we also produced large phase resettings in background activity of mPFC. Using correlation maximization criteria, we were able to estimate quantitatively the amount of phase resetting. To our knowledge, phase shifting LFPs to maximize their pair correlation was not previously used in the context of measuring the amount of phase resetting in optogentic experiments.

Although dendrogram grouping is not absolutely necessary for attractors identification, it reduced the computational time required for data analysis. For example, for *N* = 100 trials we should have performed *N*(*N* + 1) ∕ 2 ≈ 5000 pair comparisons to find if and which reconstructed phase space trajectories remained close to each other, therefore, hinting toward a possible attractor. Instead, we only checked if the individual trials from the same group remained close to each other (see red and green traces in Figures 7B1–B5). By analyzing all possible pairs of trials we would have eventually reached the same conclusion, i.e., that the individual trials from group 1 (Figure 7B1) do not remain close to the reconstructed trajectories from group 5 (see Figure 7B5).

We showed that the recorded LFPs from mPFC of ChR2 expressing PV+ interneurons could be successfully embedded in a three dimensional space. For this purpose, we presented a detailed analysis of delay embedding procedure for LFPs in response to a brief 10 ms light pulse. Both the autocorrelation and the AMI gave consistently close estimations of delay, or lag, time (see Table 1). We found that a sufficient embedding dimension was *d*_*E*_ = 3 for all six animals. The embedding dimension estimation based on the FNN method was stable for a broad range of lag times around the optimally predicted values. We also considered a wide range of values both for the ratio of the distances between neighbors in successively larger phase spaces (parameter *f* in FNN routine—see Section 4.2) and different Thieler window (parameter *t* in FNN routine).

We found the same “8”-shaped attractor, or its topologically equivalent counterparts after appropriate phase shifting, in all six animals, which covers overed 80% of recorded data. All the other attractors were produced by low-amplitude and higher frequency oscillations of LFPs, which led to a more complex structure of the attractor. One possible reason for such a clear separation into two classes of attractors across all animals could be due to neural network bistability, i.e., depending on the phase of the light stimulus the network's activity could lead to one attractor (the “8”-shaped) or a more complex geometry. Another possible, much simpler, explanation could be that the recording quality was intermittently degraded by unknown factors, such as laser trigger malfunction, etc. Future LFP recordings are required to test such hypotheses.

Additionally, the low-dimensional attractor that we identified opens the possibility of fitting the experimental data to a three-dimensional model for the purpose of better understanding the dynamics of the network, e.g., through bootstrap method (Efron, 1982).

## 7. Conclusions

The activity of medial prefrontal cortex of six optogenetic mice was periodically perturbed with brief laser pulses. The pair correlations between recorded LFPs were enhanced by appropriate phase shifting them to account for the light-induced phase resetting of network activity. The phase space dynamics was reconstructed using delay embedding method. We found that the reconstructed attractors are three dimensional and they have similar shapes across different animals.

## Author contributions

SO tested data nonlinearity, corrected data for phase resetting using crosscorrelation, computed dendrogram-based statistics, carried out numerical simulations for delay-embedding, and wrote the manuscript. PL contributed to delay-embedding numerical simulations. TT and AL performed the experiments and reviewed the manuscript.

## Funding

SO acknowledges support for this research from NSF-CAREER award IOS 1054914 and MUSC bridge funding (AL).

### Conflict of interest statement

The authors declare that the research was conducted in the absence of any commercial or financial relationships that could be construed as a potential conflict of interest.
