# Supplementary material for: Low-dimensional attractor for neural activity from local field potentials in optogenetic mice
Source: Front Comput Neurosci. 2015 Oct 2;9:125. doi: 10.3389/fncom.2015.00125 (PMC4591433; doi:10.3389/fncom.2015.00125)
Supplement: Supplementary file 3 [file Presentation1.PDF]

Supplementary Material: Low-dimensional attractor for neural activity from local field potentials in optogenetic mice

Sorinel A. Oprisan*, Patrick E. Lynn, Tamas Tompa, and Antonieta Lavin
*Correspondence:
Author Name: Sorinel A. Oprisan
oprisans@cofc.edu

1 SUPPLEMENTARY FIGURES

As with any projection of a three-dimensional object on the two-dimensional printed page, it is difficult to clearly distinguish some relevant details. For example, all reconstructed attractors shown in the main text in Fig. 7 and Figs. 8-13 are three dimensional objects. However, due to the limitations of the two-dimensional projections on the printed page it looks like the phase space trajectories self-cross (see, for example, Fig. 7b1 in the main test). In order to better visualize the structure of the reconstructed attractor and convince the reader that the attractor unfolded correctly in three dimensions, we also provided movies of the rotating frame of reference.

We also provided a movie of the rotating reference frame showing an individual trial and one of its 100 surrogates after phase space reconstruction. Figure 5c of the main text shows that all surrogates require a larger embedding dimension than the original data. This in turn translates in potential self-crossings of reconstructed surrogates in a too low (three dimensional) space. As the movie shows, there are indeed some self-crossings for the reconstructed surrogate data.

Supplementary Figure 1. (a) A three dimensional reconstructed attractor in a rotating frame gives access to different viewing angles that clearly show no self-crossings of phase space trajectory. The red and green traces are two randomly selected trials from the respective group. The hypothesis that there is an attractor of steady LFP activity is supported by the fact that individual trials (red and green curves) remain close to each other al all times. The thick blue trace is the reconstructed average of the trials in the respective group, which was only provided as a visual cue to help us gauge if the reconstructed traces from individual trials remain close to each other. (b) 3D reconstructed attractor for a randomly selected trial (blue) and one of its 100 surrogates. Since the surrogates require a higher dimensional space for correct unfolding, we notice some self-crossings.
